# Implementation of new technologies designed to improve cervical cancer screening and completion of care in low-resource settings: A case study from the Proyecto Precancer

**DOI:** 10.21203/rs.3.rs-3093534/v1

**Published:** 2023-07-07

**Authors:** Sarah D. Gilman, Patti E. Gravitt, Valerie A. Paz-Soldán, Proyecto Precancer Study Group

**Affiliations:** George Washington University; University of Maryland School of Medicine; Tulane University School of Public Health and Tropical Medicine; multi-institutional

**Keywords:** Implementation Science, Cervical Cancer, Systems-Thinking, Participatory Research, Per&uacute

## Abstract

**Background:**

This case study details the experience of the Proyecto Precancer in applying the Integrative Systems Praxis for Implementation Research (INSPIRE) research methodology to guide the co-development, planning, implementation, adoption, and sustainment of new technologies and screening practices in a cervical cancer screening and management program in the Peruvian Amazon. We briefly describe the theoretical grounding of the INSPIRE framework, the phases of the INSPIRE process, the activities within each phase, and the RE-AIM outcomes used to evaluate program outcomes.

**Methods:**

Proyecto Precancer iteratively engaged over 90 stakeholders in the Micro Red Iquitos Sur (MRIS) health network in the Amazonian region of Loreto, Perú through the INSPIRE phases. INSPIRE is an integrative research methodology grounded in systems thinking, participatory action research, and implementation science frameworks such as the Consolidated Framework for Implementation Research. An interrupted time-series design with a mixed-methods RE-AIM (Reach, Effectiveness, Adoption, Implementation, and Maintenance) evaluation framework was used to examine the adoption of molecular-based primary cervical cancer screening using HPV-testing (including self-sampling), with direct treatment after visual inspection with portable thermal ablation at the primary level.

**Results:**

The participatory and system-thinking-oriented approach led to rapid adoption and successful implementation of the new cervical cancer screening and management program within 6 months, using an HPV-based screen-and-treat strategy across 17 health facilities in one of the largest public health networks of the Peruvian Amazon. Monitoring and evaluation data revealed that, within 6 months, the MRIS had surpassed their monthly screening goals, tripling their original screening rate, with approximately 70% of HPV-positive women reaching a completion of care endpoint, compared with around 30% prior to the new CCSM strategy.

**Conclusions:**

Proyecto Precancer facilitated the adoption and sustainment of molecular-based primary cervical cancer screening using HPV-testing (including self-sampling), with direct treatment after visual inspection with portable thermal ablation at the primary level and the de-implementation of existing visual inspection-based screening strategies and colposcopy for routine precancer triage at the hospital level. This case study shows how PP used implementation science approaches to guide the adoption of a new screen-and-treat strategy in the Peruvian Amazon, while facilitating de-implementation of older screening practices.

## BACKGROUND

It is not uncommon for health programs to be characterized by non-adoption, abandonment, failure to scale, or failure to integrate sustainably into practice (1). This is especially problematic, given the existing research-to-practice gap of 17 years (2). Without stakeholder engagement and program adaptation, new health programs or related innovations run the risk of experiencing poor fit to the implementation context (3). This in turn may result in barriers to acceptance, adoption, and sustainability (3). To explore and counteract these barriers, implementation science (IS) theories, models, and frameworks (TMFs), and stakeholder-engaged research approaches like systems-thinking (ST) and participatory action research (PAR) could be operationalized to increase acceptance, adoption, and sustainability of evidence-based, locally adapted screening methods, while facilitating de-implementation of technologies and systems that prove burdensome and ineffective in low-resource settings.

The focus of this case study is a strategy designed for the elimination of cervical cancer, a preventable disease that remains the 4th most common cause of female cancer worldwide, with 80% of the disease burden occurring in low-and-middle-income countries (LMICs) and higher disease burdens in low-resource settings (4). This reflects strong disparities in terms of access to care and reflects the need for adoption of effective, context-adapted prevention strategies to increase fit-to-context. In 2020, the World Health Organization launched a global strategy to accelerate the elimination of cervical cancer as a public health problem (5). For countries to be on the path towards cervical cancer elimination, the following targets should be met by 2030:

- 90% of girls fully vaccinated with the HPV vaccine by 15 years of age
- 70% of women screened with high-performance tests by 35 years of age, and again by 45 years of age
- 90% of women identified with cervical disease treated

With less than a decade remaining before 2030, the WHO’s proposed elimination goals require rapid scale-up of effective, sustainable, and context-adapted cervical cancer screening and management programs (CCSM) (6). However, in practice, sustainable implementation and adoption of these programs has been elusive, reflecting challenges inherent in implementing multilevel interventions in complex adaptive health systems subject to frequent change and competing incentives (7).

In Perú, cervical cancer is the second leading cause of cancer death among women (8). Mortality rates are especially high in the country’s rainforest region, where women are less likely to have had a Pap test than women living on the coast (9). Since 1998, cervical cancer control has been a national priority(10); however, longstanding structural barriers to Pap-based cervical cancer screening and treatment have existed throughout Perú for decades, pointing to the need for system-wide improvement that would reliably increase women’s access to screening (11).

The aim of the Proyecto Precancer (PP) research program is to facilitate the adoption of new technologies and systems for cervical cancer screening and completion of care in the Peruvian Amazon with high cervical cancer incidence rates (6). Over the past five years, PP has engaged more than 90 stakeholders from all levels of the health system in Lima (Peru’s capital), Loreto (a large region in the Amazon, in northeastern Perú), and the Micro Red Iquitos Sur (MRIS) health network (the largest health network in Iquitos—Loreto’s capital) (6).

The MRIS network covers a population of 150,000, with a target population of 20,000 women 30–49 years of age, eligible for cervical cancer screening via HPV testing. Comprised of urban, peri-urban, and rural communities, Iquitos (~ population 400K) is the largest city in the world that is accessible only by air or river. Within Iquitos, transportation routes include networks of paved and unpaved roads that connect communities to each other, one main paved highway, and boat travel by river.

Given the setting and its influence on access to health care, local champions and researchers felt that HPV-based screening (including patient self-collection) might ameliorate some of the barriers presented by cytology-based screening programs, with the added benefit of highly sensitive and objective results. Additionally, referring all positive women to the regional hospital with limited availability of colposcopy triage appointments created significant bottlenecks for continuum of care. Hence, system stakeholders agreed to manage all eligible HPV positive women at primary health facilities, with a medical professional conducting visual triage for treatment, and managing all eligible women with ablative therapy (thermocoagulation). Only women with suspected cancer, large lesions (> 75% of transformation zone), or a transformation zone that was hard to see were referred to hospital specialists. Moreover, it was clear that a top-down approach to implementation would be unlikely to yield sustainable results, and any new evidence-based interventions would need to be supported by stakeholders, adapted to the unique local-context, and owned by the health system.

The complexity inherent in making changes to the healthcare system required reflection, understanding of a new system and the engagement of multi-level stakeholders, and a guiding map for both implementers and partners. These needs led to the development of the INSPIRE (Integrative Systems Praxis for Implementation Research) research methodology, which combined ST and PAR-based approaches with existing IS models and frameworks (REFER PATTI paper). As INSPIRE was developed, PP iteratively sought to explore the following question: *“How can the use of IS models and frameworks, combined with ST and PAR approaches, influence the adoption and sustainability of new evidence-based CCSM services in a complex health system located in the Peruvian Amazon?”*

The aim of this case study is to detail PP’s use of a participatory, ST and IS-guided process with stakeholders throughout the MRIS health system – and with health authorities at regional and national levels – to guide the implementation, adoption, and sustainment of new technologies for CCSM in the Peruvian Amazon (see Fig. 1).

## METHODS

### Approaches and frameworks that informed INSPIRE

INSPIRE arose from a constellation of needs that included visualizing the health system from multiple perspectives, engaging stakeholders at all system levels, identifying barriers and facilitators to change, determining how to adapt knowledge to the local context, and planning implementation. Based on these needs, the PP decided to combine three approaches – PAR, ST, and IS -- to maximize opportunities for effective translation of evidence to practice in cervical cancer screening in this context. PAR approaches hinge strongly on stakeholder engagement, co-learning, and co-production of knowledge and potential solutions (12, 13). PP brought evidence-based CCSM innovations and concepts, but the PP research team recognized the context expertise of the stakeholders; building trust and mutual respect would increase the likelihood of context-relevant decisions that would lead to adoption and sustainment. ST can be particularly helpful for developing clear mental models of complex systems (such as health care systems) involving multiple perspectives, systems levels, and interacting agents that evolve over time (14, 15). To fully understand CCSM in this setting, as well as its constraints, barriers and facilitators ST serving as a powerful tool to enhance sense-making. PP used this approach to ensure that the health system was visualized from multiple levels and perspectives, and that stakeholders at all system levels were engaged in the identification of barriers and facilitators to CCSM.

PP leveraged IS theories, models, and frameworks (TMFs) to: 1) guide translation of HPV-based screen and treat methods from research to practice, 2) understand influences on HPV-based screen and treat outcomes, and 3) evaluate the implementation of the screen and treat program (16). Tables 1 and 2 (see Additional File 2 for Table 2) describe TMFs and approaches used in different phases of INSPIRE. These frameworks, like the Consolidated Framework for Implementation Research (3), and the RE-AIM (reach, effectiveness, adoption, implementation, maintenance) framework (17, 18, 19), helped drive the PAR and ST approaches at the core of INSPIRE in an iterative learning cycle that guided the process of knowledge creation and application. CFIR-a determinant framework-facilitated ‘sense-making’ in the project - helping researchers consider gaps or barriers in the system that would require development or adaptation to ensure proper fit to context. For example, the lack of an individual-level monitoring and evaluation system led to the development of a hybrid electronic-paper registry.

INSPIRE: Integrative Systems Praxis for Implementation Research consists of four phases, with specific implementation strategies to meet the objectives of each phase.

The RE-AIM framework was selected to guide evaluation, by defining key indicators to monitor over time. We include additional information about the IS frameworks used in Additional File 1.

### The INSPIRE Model

Bringing these approaches and frameworks together, INSPIRE consists of four key phases:

- Phase 1: Understand the system
- Phase 2: Find leverage
- Phase 3: Act strategically
- Phase 4: Learn and adapt

Each phase of INSPIRE (see Fig. 2) relies on defined implementation strategies to meet its objectives.

### Case Study Methods by INSPIRE Phase

In Phase 1, interviews and focus groups with different system stakeholders were conducted to generate a detailed visual of the system and all its complexity from multiple perspectives. By creating a visual of the CCSM system, PP moved stakeholders from “solving” or “judging” parts of the system to “understanding” that all were part of a complex system with multiple barriers: fragmentation, duplication, delays, insufficient training among health professionals on screening and triage techniques, and lack of a monitoring system. The visualization of the system from multiple perspectives enabled a shared understanding of current and ideal systems to begin to emerge, but also led stakeholders to comprehend that all parts of the CCSM mental model were necessary for the system to work effectively: for example, by acknowledging that screening without clinical follow up was useless.

Figures 3 and 4 illustrate mental models of an idealized CCSM system, as well as a more realistic mental model, with key considerations that resulted from the INSPIRE process.

In Phase 2, we engaged multilevel stakeholders in group model-building workshops (20), sharing, testing, and revising system maps, and then defining and localizing problem areas, including bottlenecks, fragmentation, and duplication. The group model building enabled us to ultimately find leverage for change. In these group model-building workshops, PP chose to focus specifically on a combination of deliberative dialogue (21) and scenario analysis (22, 23, 24), in order to test assumptions in real-time, using a collaborative, exploration-focused approach that helped to bring about a shared vision for future implementation. In scenario analysis, stakeholders and researchers examined simple models of several evidence-based CCSM approaches. Specifically, based on assumptions that could be modified in real-time, stakeholders could observe the predicted trade-offs of various approaches (i.e., observing how one screening or triage decision could affect the total number of women with precancer or cancer who are ultimately identified or treated), helping them to define a preferred CCSM approach for adoption and evaluation. Figure 5 shows a real-world example of output from the scenario analysis tool, used in one of the design workshops.

Stakeholders ultimately decided on the CCSM approach that would be adopted in Iquitos and were confident about this decision due to the shared knowledge about evidence and options available to them. In Phase 3, PP focused on strategic action, developing stakeholder-designed implementation plans (via stakeholder working groups), conceptualizing infrastructure modification (from adapting an internal hybrid monitoring and evaluation system to evaluate impact, to working with authorities to add new services to the public health billing system, to adapting laboratory space for sample processing), and helping the health system to prepare for task-shifting and training health care providers (including lab technicians, midwives, medical doctors at the primary level, and specialists). Additional strategic actions included developing dissemination plans and planning key program adaptations. Across all phases, but especially in phase 3, PP bundled discrete implementation strategies to meet stakeholder needs across the implementation continuum (acceptability, adoption, implementation, scale-up, and maintenance). For example, three working groups independently met to plan for changes at the primary screening level, for thermocoagulation, triage, and treatment, and for hospital-level referrals and management. Each working group produced a plan describing, not just what activities should be completed at every phase but by whom and how they should be completed.

In Phase 4, the focus was on deliberative dialogue to establish ongoing monitoring and evaluation using stakeholder-accepted outcomes, followed by review of monitoring and evaluation results with stakeholders, and re-initiation of the INSPIRE cycle where indicated. For example, when screening increased significantly in urban health facilities but not the rural health facilities, key stakeholders met to discuss the range of challenges and potential solutions (phase 1), find leverage in the system (phase 2), and to plan and implement possible solutions and make adaptations (phase 3). In this example, community campaigns in rural areas with self-collection were undertaken to reach women in their homes.

### Intervention, Adaptation, and Implementation Strategies

From March 2017 to June 2019, the project focused on INSPIRE phases 1–3. This included interviews and system audits, group model-building workshops with stakeholders throughout the health system, establishing a patient-level screening registry, implementation planning workshops, and preparation for implementation of the CCSM program among women ages 30–49, eligible for HPV-based testing. Table 2, attached as a supplemental file, lists the research methods, IS frameworks, and implementation strategies used, by phase of INSPIRE (6). Phased implementation of the new CCSM strategy (de-implementing VIA and Pap while implementing HPV testing and thermoablative treatment) took place from July 2019 to the present, albeit with interruptions due to COVID-19 and supply-chain issues with testing supplies. Evaluation of the program (Phase 4 or “learn and adapt”) is ongoing to contribute to the iterative improvement of the program.

Program adaptation to ensure fit with the local context was critical to adoption. Prior to PP, health professionals filled out numerous paper-based forms for billing, clinical records, and patient follow-up. The PP team facilitated the design of a triple-copy form that could meet the multiple documentation needs of the health system, but that could also be used for the hybrid electronic-paper patient-level screening registry to ensure adequate tracking of cases and reduction of loss to follow up. The de-implementation of Pap-smear and VIA as a screening method, and the introduction of HPV testing that included patient self-collection also required new nurse-midwife trainings and new approaches to health counseling. Additional adaptations included modifying Ministry of Health billing practices to allow for new procurement processes related to CCSM purchases and supplies, modification of clinical forms for data and patient monitoring, and changes to the insurance reimbursement process. The INSPIRE process also led to the differential adaptation of the screening process in rural areas by delivering services using campaign-style methods, as opposed to more routinely available services in urban or peri-urban areas.

### Measurement & Design

This implementation research study used an interrupted time series design with a mixed-methods RE-AIM (Reach, Effectiveness, Adoption, Implementation, and Maintenance) evaluation framework. RE-AIM was selected due to its strong evidence base in program evaluation, while qualitative research augmented evaluation findings through the exploration of additional factors of importance to stakeholders.

### Reach

Reach was assessed quantitatively, by estimating coverage targets as monthly unique screening tests in women aged 30–49 years old, based on the WHO goal of encouraging screening for 70% of 20,000 over a 3-year (VIA) or 5-year (HPV) period (n = 389/month and 233/month, respectively). The percent of monthly target screening tests was tracked using visual inspection with acetic acid and compared to the percent of participants screened using HPV-based testing. Change in screening rates in the pre-implementation time period (Jan 1, 2018-Jun 30, 2019) compared with the post-implementation time period (July 1, 2019 – Feb 27, 2020) was then evaluated in an interrupted time series analysis with Poisson regression, modeling screening counts and adjusting for seasonal variation. Fourier terms and a scaling parameter were included to allow for over-dispersion of data.

### Effectiveness

Effectiveness was evaluated by tracking the proportion of women who completed the continuum of care for CCSM, comparing the proportion of Pap/VIA screen-positive women who attended colposcopy with the proportion of HPV screen-positive women receiving thermoablative treatment for management of precancerous lesions, or referral to colposcopy if ineligible for ablation. Additionally, semi-structured interviews were conducted with stakeholders at all levels of the health system, including patients completing care, those who did not complete the continuum of care, and staff delivering the program.

### Adoption

To measure adoption, PP collected data on the proportion of health facilities that utilized HPV-based testing methods to screen women ages 30–49. Focus groups and in-depth discussions were also held with midwives classified as high and low HPV testing adopters. Proportion of total screens for VIA as compared to HPV-based testing were also compared over time. Additional key considerations that were highlighted by stakeholders and explored through workshops, focus groups, and in-depth interviews were acceptability, appropriateness, costs, feasibility, fidelity, penetration, and sustainability.

### Implementation

Implementation measures focused on time and motion studies, evaluating time from sample collection to testing, result delivery to patient, and result delivery to follow-up visit. Qualitative research was also conducted to evaluate quality of patient care throughout the care continuum.

### Maintenance

To evaluate potential for maintenance, PP assessed changes in reach and effectiveness measures over time and qualitatively noted any events in the broader context that might influence or disrupt time trends (for example, supply chain disruptions, COVID-19, or political turnover).

The Standards for Quality Improvement Reporting Excellence guidelines were used as a framework for reporting this case study (25). A table mapping the guidelines to each section of the manuscript is included below in Additional File 3 (SQUIRE checklist).

## RESULTS

### Stakeholder Engagement

More than 90 stakeholders throughout the Micro Red Iquitos Sur health network collaborated with PP throughout INSPIRE. Key stakeholder groups included Peruvian and international researchers, patients, primary health care providers, gynecologists, midwives, nursing assistants, laboratory staff, administrative staff, and representatives from the Peruvian Ministry of Health (MINSA), the Regional Ministry of Health (DIRESA) in Loreto, the National Institute of Neoplastic Diseases, the National University of the Peruvian Amazon (UNAP), the National Association of Psycho-Oncology of Peru (ANNPPe), the Pontifical Catholic University of Perú (PUCP), and the Escuela de Excelencia en Consejería (“School of Excellence in Counseling”). Co-developed stakeholder knowledge and strategies emerged through process mapping, in-depth interviews, focus groups, and program audits (Phase 1); group model building, deliberative dialogue, and scenario analysis (Phase 2); designing implementation plans, infrastructure modifications, training, and dissemination plan development (Phase 3); and ongoing monitoring and evaluation, as well as iterative co-learning and improvement (Phase 4).

### Reach

PP monitoring and evaluation (M&E) data showed that almost 3,000 HPV screening tests were collected among the primary intervention target group (non-pregnant women aged 30–49 years), with approximately 20% of these (602) positive for HPV (26). Among the primary intervention target group (non-pregnant women aged 30–49 years), PP M&E data showed that screening rates surpassed 70% monthly coverage targets established according to WHO’s elimination goals within 6 months. In comparison, prior to HPV testing, only 31% of women were being routinely screened with either Pap or VIA (26, 27).

### Effectiveness

PP precancer monitoring and evaluation data show that a completion of care endpoint was reached by 67.4% (406/602) of HPV-positive women within 6 months, compared with 30.2% (52/172) of VIA-positive women at hospital facilities (p < 0.001) (26). Figure 6 (pre-implementation) and Fig. 7 (post-implementation) compare pre-and-post-implementation results for completion of care and loss to follow-up.

### Adoption

The HPV screen-and-treat intervention demonstrated rapid adoption within 6 months across 14/17 health facilities in the Micro Red Iquitos Sur (MRIS) health network; in the same time period (July 2019-November 2019), VIA screening was de-implemented in all 17 primary health facilities (6). Moreover, in addition to screening, two health facilities served as thermocoagulation centers. One health facility offered thermocoagulation on a daily basis to HPV + women who were eligible and had been referred to them, and the other health facility offered a thermocoagulation clinic on Thursdays. This was an example of local adaptation, with each health facility making different decisions based on its own unique needs and contexts. One of these teams later began offering travel clinics, meeting women at rural health clinics to circumvent structural barriers to screening posed by geographic and transportation challenges. PP interpreted these local and continued adaptations as evidence of health-system ownership and program success.

### Implementation

Time and motion studies found that the median time per participant at in-clinic HPV screening visits was 3 hours and 6 minutes (from arrival to departure) (28). HPV test results were available within a median of 7 days (interquartile range 4–13) and median time spent at the visual assessment with ablative treatment was 2 hours and 40 minutes (from arrival to departure) (28). In contrast, the median time for delivery of pap results was 20 days. HPV sample collection and ablative treatment required only a median of 5 minutes and approximately 20 minutes for registration and counseling per patient, indicating the potential to reduce wait times (28). While the time and motion studies indicate that improvements are needed, they also demonstrate how this type of data can be used to iteratively improve system learning, planning, and sustainment, especially when paired with simulation models and ongoing quality improvement discussions (28, 29).

Qualitative implementation data was also collected around women’s experiences with self-collection, and before, during, and after treatment with ablative therapy, with findings indicating strong support for both modalities (30). Women found both self-collection and ablative therapy to be highly accessible and acceptable: up to 90% of women in rural areas self-collected, compared to approximately 60% in urban areas, and all (100%) women who had the choice of visual triage to determine eligibility for thermocoagulation at the primary level versus or seek a specialist referral to the hospital chose visual triage at the primary level. Additionally, participants who received thermocoagulation stated emotional satisfaction of a swift and accessible resolution at local primary health facilities (30). Interviews and time and motion data, when combined with the quantitative data on reach and adoption, revealed that task-shifting management of screen-positive women to primary health facilities significantly improved quality of care, reduced loss to follow up and improved completion of care.

### Sustainability

Regional support for the program remains high and includes the organization of stakeholder-driven adaptation workshops and a regional proclamation by policymakers, prioritizing the regional reduction of cervical cancer. Through political upheaval (Perú has had six presidents during this time period) and one instance of pressure to return to the previous pap-based screening mode (over a period of six months), regional authorities and health professionals in Iquitos have continually asserted that the HPV-screen-and-treat strategy is most feasible for their context.

The HPV screen-and-treat program in Iquitos, Peru was impacted by, but ultimately resilient to, pandemic service disruptions, political instability, flooding, health system strikes, and supply chain disruptions, suggesting a high probability of sustainability over time. Iquitos was one of the world regions hardest hit by COVID-19 between March and June 2020, with a COVID-19 prevalence of 70% by July 2020, despite lockdowns due to COVID-19 (31). The CCSM program was paralyzed for three to four months, but resumed fully by August 2020, without outside intervention.

Using evidence generated by this program, the Peruvian Ministry of Health is scaling-up beyond the Micro Red Iquitos Sur health network to the entire state of Loreto, and to 14 new regions of Perú located in the Amazon, on the coast and in the mountains, using this NCI-funded Iquitos experience as a national pilot.

## DISCUSSION

Through visualization of the health system and the processes and interrelationships that influence it, PP facilitated adoption of self-sampling, molecular-based primary cervical cancer screening, and direct treatment (after visual assessment) with portable thermal ablation. This includes the de-implementation of existing visual inspection-based strategies and colposcopy for routine precancer diagnosis.

This case study underscores the scientific relevance of employing IS approaches in global health, in conjunction with ST and PAR, to help us reach the WHO’s cervical cancer elimination goals, by adopting, implementing, and adapting feasible and effective screening and management strategies while facilitating de-implementation of less feasible approaches, and creating a more equitable health system for women. Using the INSPIRE methodology to engage the implementers in implementation research and IS TMFs for sensemaking of a complex and dynamic implementation experience allowed us to bridge implementation research and practice in a meaningful way.

Program champions and key opinion leaders were of critical importance to the adoption and sustainment of new CCSMs: their knowledge of their context and how to work and create change within the system was key. Moreover, this program offers a good example of how academic-public health system partnerships can facilitate better implementation planning at regional and national levels. For example, PP had the resources and capacity to evaluate the relative effectiveness of specific strategies that the Ministry of Health was concerned about, but for which they needed local evidence. Likewise, PP knew that sustainability was only possible if the public health system adopted and felt ownership around the intervention.

We have also observed important synergies between stakeholder engagement and audit/feedback strategies that are supported when using interrupted time series in LMIC contexts, as opposed to randomized designs which may distance stakeholders from a more unconstrained, real-world context, limiting the amount of actionable feedback that can be obtained. The use of qualitative research was especially useful as PP’s research team sought to understand how and why specific outcomes were observed.

In large part, the use of IS concepts was driven by stakeholder desire to reduce health disparities, increase access to care, and ensure the sustainability of the new CCSM services. Stakeholder engagement in the selection of several alternative evidence-based screening and management strategies led to a system that is more equitable. In the initial phases of INSPIRE, a question asked of stakeholders was: “What is the most feasible, evidence-based, and equitable CCSM that can be offered in this context?”. The ensuing discussion revealed that, through the reduction of systemic barriers, HPV-based screening combined with follow-up of thermal ablation at the primary level for those who are eligible and referral to hospital of only those at greatest risk would avert a far greater number of cervical cancer cases, increasing the number of women able to successfully access the health system.

The results observed in this case study have been actively disseminated through presentations, key partnerships, trainings, discussions, and engagement of key opinion leaders, as well as through passive diffusion amongst professional, regional, and digital networks. More data is needed regarding the mechanisms that influenced stakeholders to adopt these new CCSM practices, and how these practices may become embedded into routine practice. We are employing Normalization Process Theory (32), a sociological theory and IS framework, to explore how providers perceive the changes to the health care system and have found it useful in identifying what changes to implementation might result in improved adoption and sustainment. We are also conducting a realist evaluation to identify the underlying mechanisms that led to change (33). We continue to measure loss-to-follow-up rates among HPV-positive women and are exploring why 30% of women – mostly women referred to the hospital – are still not making completion of care, so that we can improve on these findings (34). Given that this is a challenge encountered by other screen-and-treat programs in Latin America, we are eager to share our own localized understanding of why this particular barrier exists and how it may be mitigated. Given the nature of this research, and the unique implementation context, this case study cannot be generalized to all cervical cancer screen-and-treat programs. However, we hope this case study will serve to provide helpful information and examples for implementers in similar contexts.

## CONCLUSIONS

Proyecto Precancer applied the INSPIRE framework to describe, guide, and analyze the implementation experience. This case study contributes to the existing body of knowledge around cervical cancer prevention by showing that task-shifting strategies to primary care were feasible and effective in this context and that the knowledge-sharing and shared decision-making enabled by the program likely resulted in higher ownership over the strategy, increasing the likelihood of positive and sustainable change.

Through a renewal grant funded by the National Cancer Institute, PP is developing a multi-faceted implementation strategy and toolkit to facilitate the scale-up of context-adapted HPV-based CCSM programs in diverse contexts, including throughout Perú, and possibly Latin America.

We strongly believe that the use of IS and other interdisciplinary research approaches will generate evidence that enables rapid scale-up of effective and context-adapted CCSM programs, ultimately contributing to the global elimination of cervical cancer as a public health problem. Evidence-based interventions will offer partial solutions to help reach the WHO’s proposed elimination goals, but the most reliable progress will be achieved through the judicious and long-term use of IS strategies to drive adoption, sustainment, and scale-up of evidence-based practices within locally-adapted strategies and programs.

## Figures and Tables

**Table 1:** Approaches, models, and frameworks that help explain the mechanisms behind INSPIRE.

Name	Type	Purpose
*Consolidated Framework for Implementation Research (CFIR)*	Determinant	To classify barriers and facilitators to implementation in a multi-level, complex health system that surfaced during the INSPIRE methodology, allowing appropriate matching of implementation strategies to context and more generalizable reporting of results
*RE-AIM (reach, effectiveness, adoption, implementation, maintenance) Framework*	Evaluation	To assess the potential influences that INSPIRE had on CCSM practices throughout the MRIS health network
*Systems Thinking*	Theoretical Approach	To converge stakeholder mental models of complex health care systems involving multiple perspectives to facilitate shared decision making, action, reflection and adaptation
*Participatory Action Research*	Action Research Approach	To encourage stakeholder engagement, co-learning, and co-production of knowledge

**Table 2:** Integration of research methods, implementation science (IS) frameworks, and implementation strategies by INSPIRE phase

	INSPIRE action	Research methods utilized	IS Frameworks	Implementation strategies
INSPIRE HUB	1. Define problem situation with stakeholders 2. Launch the project	Soft systems methodology	*CFIR Domain 1:* intervention source *CFIR Domain 5:* engaging	Build buy-in (involve existing governance structures, ID champions) Develop relationships (build coalitions, resourcesharing agreements, formal commitments, academic partnerships)
Phase 1: UNDERSTAND THE SYSTEM	3. Develop mental models of the system 4. Establish narrative and stakeholder perceptions of the system. 5. Make the system visible	AIIM SAST stage 1 with key informant interviews and focus group discussions KAP surveys Audits of current system outcomes Pathway analysis visually represented by flow charts and swim-lane diagrams	*CFIR Domain 3:* defining structural characteristics, networks, & communications *CFIR Domain 2:* culture & implementation climate *CFIR Domain 3:* patient needs and resources *CFIR Domain 3:* external policies & incentives *CFIR Domain 4:* knowledge & beliefs about the intervention *CFIR Domain 4:* understand selfefficacy, individual stage of change and other attributes	Gather information (needs assessment, readiness to change) Involve patient/consumers and family members Audit current system behavior Capture and share local knowledge
Phase 2: FIND LEVERAGE	6. Engage stakeholders in group model building 7. Share, test, revise system/process maps 8. Define and localize system behaviors contributing to problem situation 9. Find leverage for change	SAST Stage 2 – design workshops Dialectic debate and group model building (facilitated with goal to balance desirability and feasibility guided by reflection on implementation outcomes such as feasibility, cost, acceptability, sustainability, etc). Scenario analysis	*CFIR Domain 1:* review characteristics of the intervention and options (evidence strength and quality, relative advantage, complexity, cost) & assess adaptability and trialability of alternatives *CFIR Domain 3:* assess cosmopolitanism, peer pressure, influence of external policies/incentives *CFIR Domain 4:* assess KAB about intervention options *CFIR Domain 4:* Group level stage of change	Assess readiness and identify barriers Get feedback from audit of current system behavior Purposefully reexamine the intervention Tailor strategies to overcome barriers and honor preferences Model and simulate change Conduct local consensus discussions Distribute educational materials and conduct educational meetings Make training/education dynamic and participatory Inform local opinion leaders Create a learning collaborative Consider restructuring strategies as leverage opportunities Consider financing strategies as leverage opportunities Mandate change
Phase 3: ACT STRATEGICALLY	10. Stakeholder designed implementation plan 11. Infrastructure modifications, training, dissemination plan development 12. Implement changes	Work group soft systems methodology with research team facilitation	*CFIR Domain 1:* design quality *CFIR Domain 1:* complexity *CFIR Domain 5:* planning *CFIR Domain 5:* executing	Develop a formal implementation blueprint Tailor strategies to overcome barriers and honor preferences Stage implementation scale-up Involve patients/consumers and family members Recruit, designate, and train for leadership Obtain formal commitments Develop effective educational materials relevant to mandated change Develop a glossary of implementation (including new models) Distribute educational materials Conduct ongoing, dynamic training Conduct educational outreach visits Use train-the-trainer strategies Provide ongoing consultation Place new interventions on fee for service lists/formularies *Develop supply chain management* Revise professional roles Create new clinical teams Change services sites Change equipment Change records systems Develop and organize quality monitoring systems Develop tools for quality monitoring Use advisory boards and work groups Conduct cyclical tests of change Create or change credentialing and/or licensure standards
Phase 4: LEARN AND ADAPT	13. Ongoing M&E using stakeholder defined implementation outcome metrics 14. Share M&E with stakeholder group 15. Re-initiate INSPIRE cycle where indicated by identification and localization of new or unresolved problem situation	M&E for primary implementation outcomes SAST with KII and FGD Design workshops	*RE-AIM* *CFIR Domain 5:* Reflecting and evaluating	Provide ongoing consultation Sustain a learning collaborative Use mass media to increase reach *(only after system behavior is stabilized postimplementation)* Use advisory boards and working groups Organize clinical implementation team meetings

Reproduced from *Cancer Epidemiology, Biomarkers & Prevention*. 2020. Vol.29,9, 1710–1719, Gravitt, P. et al, “Integrative Systems Praxis for Implementation Research (INSPIRE): An Implementation Methodology to Facilitate the Global Elimination of Cervical Cancer., with permission from the American Association for Cancer Research (AACR).

[Table 2 will be included as an additional file]

## Abbreviations

CCSM: Cervical Cancer Screening and Management
IS: Implementation Science
MOH: Ministry of Health
MRIS: Micro Red Iquitos Sur
PP: Proyecto Precancer
TMFs: Theories, Models, and Frameworks
VIA: Visual Inspection with Acetic Acid
